# Endoscopic removal of high‐powered magnets from the appendiceal orifice in an asymptomatic child

**DOI:** 10.1002/jpr3.12168

**Published:** 2025-02-28

**Authors:** Jeanette Freeman, Steven D. Miller, Brett J. Hoskins

**Affiliations:** ^1^ Department of Pediatrics The Johns Hopkins University School of Medicine Baltimore Maryland USA; ^2^ Division of Pediatric Gastroenterology, Hepatology, and Nutrition, Department of Pediatrics The Johns Hopkins University School of Medicine Baltimore Maryland USA; ^3^ Division of Pediatric Gastroenterology, Hepatology, and Nutrition, Department of Pediatrics Indiana University School of Medicine, Riley Hospital for Children at IU Health Indianapolis Indiana USA

**Keywords:** endoscopy, foreign body appendicitis, magnet ingestion, pediatrics

## Abstract

Ingestion of multiple magnets can lead to serious complications, including foreign body appendicitis. Appendicitis usually develops when an object blocks the appendiceal orifice, though outcomes may vary from asymptomatic passage to acute inflammation. While several case reports have documented appendectomy for magnet‐induced foreign body appendicitis, and one report described endoscopic removal of magnets in a patient with appendicitis, this case is the first to report successful endoscopic removal of high‐powered magnets from the appendix in an asymptomatic child. This intervention potentially prevented the development of appendicitis and the need for surgery. This case highlights the importance of considering foreign body retention in the appendix when objects fail to progress beyond the right lower quadrant.

## 1 INTRODUCTION

Oral exploration is a typical part of childhood development but carries the risk of ingesting foreign bodies, including high‐powered magnets. These magnets, typically made from rare‐earth materials like neodymium, can exert attractive forces more than 5 times stronger than conventional magnets.^1^ The incidence of high‐powered magnet ingestion in children has risen at an alarming rate over the past 15 years.^2^ While a single small magnetic object will often pass through the gastrointestinal tract without issues, ingesting more than magnet may lead to severe complications like intestinal obstruction, ischemia, necrosis, fistula formation, perforation, and appendicitis.^3^, ^4^ The presence of sodium, potassium, and chloride ions in human cells makes the tissue conductive, enabling interaction with magnetic forces and increasing the risk of magnetic foreign bodies eroding through the bowel.^3^ A high index of suspicion is required for foreign bodies that persist in the right lower quadrant on serial imaging, as these may be lodged in the appendix, where they are difficult for the body to expel into the large intestine.^5^ Here, we describe a case of a 10‐year‐old boy who underwent successful endoscopic removal of high‐powered magnets from the appendix, avoiding potential development of foreign body appendicitis and need for surgical intervention.

## 2 CASE REPORT

**Figure 1:**
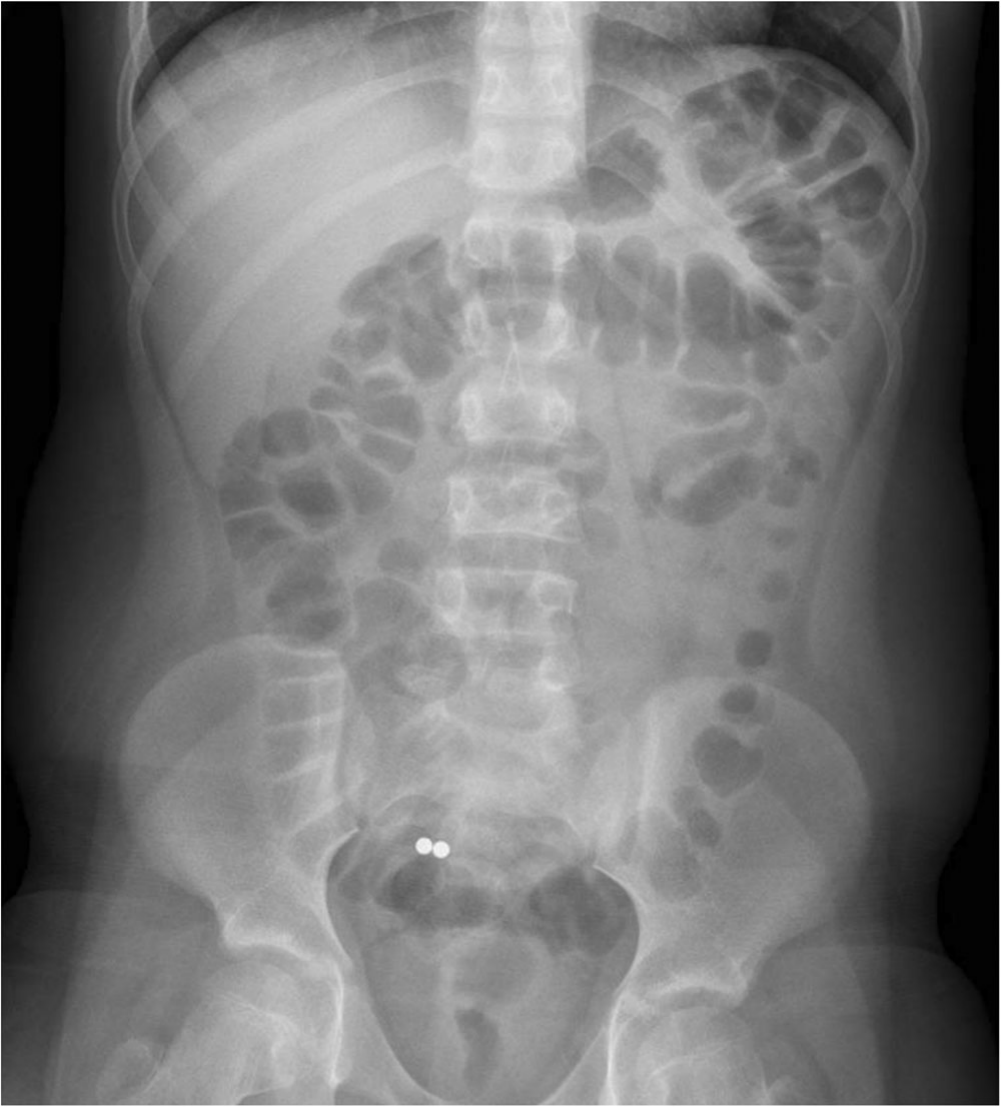
Image from abdominal X‐ray showing circular foreign bodies in the right‐lower quadrant.

A 10‐year‐old boy with a history of attention‐deficit/hyperactivity disorder and seasonal allergies presented after an unwitnessed ingestion of two high‐powered magnets. At an outside hospital, radiography revealed two small, circular foreign bodies in close proximity within the stomach, consistent with connected high‐powered magnets. During transfer to our institution, the objects had moved into the small intestine, and were unable to be obtained via esophagogastroduodenoscopy. Radiography suggested both objects had remained attached. Following the management algorithm proposed by Kramer et al.,^1^ the patient was placed on a clear liquid diet and underwent an oral bowel cleanse with polyethylene glycol, while his stool was closely monitored for passage of the objects. Stimulant laxatives were not used. Serial radiographic images were performed, showing the two objects remained together in the right‐lower quadrant (Figure 1). Given lack of progression from this area for two days, colonoscopy was performed.

**Figure 2:**
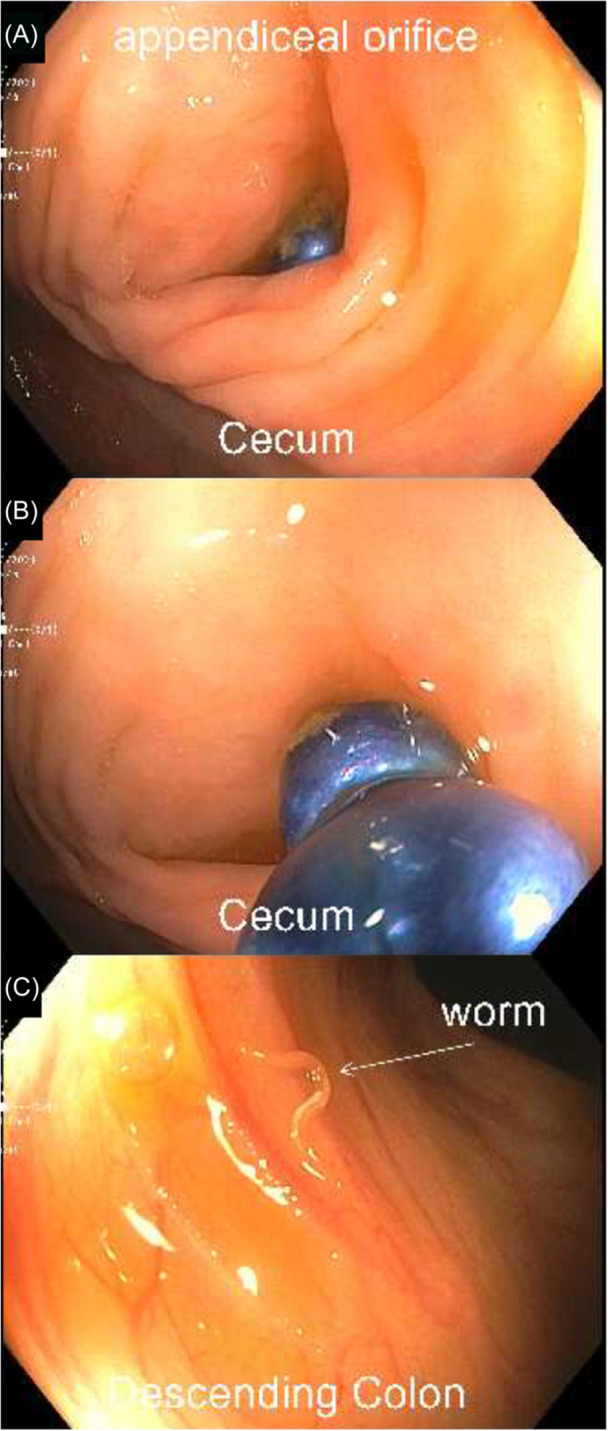
Endoscopic images during colonoscopy showing successful removal of 2 high‐powered magnets from the appendiceal orifice.

One of the small, circular magnets was partially visible in the appendiceal orifice during colonoscopy (Figure 2A). Tripod forceps were used to grasp and secure the visible magnet, which exhibited strong attractive forces, remaining attached to both the forceps and the other magnet. Both magnets were successfully removed together using this technique (Figure 2B). Repeat imaging at the end of the case confirmed there were no additional foreign bodies present. Motile pinworms had been incidentally found during the colonoscopy (Figure 2C), and he was treated with albendazole. Post‐procedurally, he tolerated diet advancement and was discharged home with no additional interventions. There was no indication of acute appendicitis based on physical examination, complete blood counts, inflammatory markers, or abdominal ultrasonography throughout the hospitalization. This case was subsequently reported to the United States Consumer Product Safety Commission.

## 3 DISCUSSION

While foreign body appendicitis often occurs shortly after an object enters the appendiceal orifice, outcomes vary, ranging from acute appendicitis two years after object ingestion to asymptomatic passing of a foreign body from the appendix two months after ingestion without appendicitis.^6^ Factors that may influence development of foreign body appendicitis include individual anatomy, number of objects ingested, and the object's size, shape, and material. Multiple case reports describe appendectomy for foreign body appendicitis due to high‐powered magnets, and one case describes endoscopic removal in a patient with appendicitis.^7^

One case report presented a 5‐year‐old boy without significant past medical history who ingested two pennies and four magnets. The patient received high‐dose polyethylene glycol, bisacodyl, and enemas with serial imaging to monitor progression. While the pennies passed, the magnets remained connected in the right‐lower quadrant after two days. Multiple unsuccessful attempts were made at endoscopic removal, and he required a laparoscopic exploration, ultimately resulting in an appendectomy and partial cecal resection.^8^

An additional case report described a 23‐month‐old girl who ingested a chain of 11 connected magnets. This patient presented to the emergency department after two days of intractable vomiting with interval development of coffee‐ground emesis, fatigue, irritability, tachycardia, moderate dehydration, leukocytosis, and thrombocytosis. She was started on a bowel cleanout in anticipation for likely surgical intervention. The connected magnets were successfully removed from the appendix during colonoscopy with endoscopic forceps, and she was treated with a course of oral antibiotics for acute appendicitis.^7^ While this patient underwent successful endoscopic removal, she presented with evidence of acute appendicitis, and with a high index of suspicion surgical intervention would be required. In contrast, our patient was well‐appearing, without vomiting, dehydration, or abnormal laboratory findings.

To the best of our knowledge, this is the second report describing successful endoscopic removal of high‐powered magnetic foreign bodies from the appendiceal orifice in children or adults, but the first in a patient before development of appendicitis. Foreign bodies are a rare, but important, cause of acute appendicitis in both children and adults. Ingestion of multiple high‐powered magnets may result in ischemia, necrosis, perforation, and fistula as a result of the object's attractive forces across multiple layers of bowel and the conductive nature of human tissue. Maintaining a high index of suspicion that an object may be in the appendix is important when magnetic foreign bodies do not progress from the right lower quadrant on radiography. In these cases, early endoscopic removal may potentially reduce the risk for development of foreign body appendicitis and need for surgical intervention.

## CONFLICT OF INTEREST STATEMENT

The authors declare no conflicts of interest.

## ETHICS STATEMENT

Informed written consent for case report publication was obtained from the patient's parents before article submission.

## References

[1] Kramer RE , Lerner DG , Lin T , et al. Management of ingested foreign bodies in children: a clinical report of the NASPGHAN Endoscopy Committee. J Pediatr Gastroenterol Nutr. 2015;60:562‐574. 10.1097/MPG.0000000000000729 25611037

[2] Alansari AN , Baykuziyev T , Soyer T , et al. Magnet ingestion in growing children: a multi‐center observational study on single and multiple magnet incidents. Sci Rep. 2024;14:4575. 10.1038/s41598-024-55127-0 38403623 PMC10894856

[3] Zefov V , Hashemi HA , Javaid U . Accidental ingestion of magnetic foreign body in a pediatric patient: a potentially fatal attraction. Radiol Case Rep. 2022;17:2337‐2341. 10.1016/j.radcr.2022.04.007 35570859 PMC9095658

[4] Liu S , Li J , Lv Y . Gastrointestinal damage caused by swallowing multiple magnets. Front Med. 2012;6:280‐287. 10.1007/s11684-012-0207-5 22886320

[5] Lee M , Kim SC . Appendiceal foreign body in an infant. Medicine. 2017;96:e6717. 10.1097/MD.0000000000006717 28445284 PMC5413249

[6] Hamadneh M , Al‐Khalaileh M , Alayed A , et al. Previous foreign body ingestion in the appendix causing acute appendicitis: a case report. Cureus. 2023;15:e34948. 10.7759/cureus.34948 36938180 PMC10017911

[7] Nazzal K , Nazzal O , Ahmed A , Alaradi H , Alhindi S . Magnet beads impacted in the appendix of a child: a case report and review of the literature. Cureus. 2020;12:e9777. 10.7759/cureus.9777 32953295 PMC7491694

[8] Birkhold M , Habib JR , Kang J , Diaz‐Calderon L , Lumpkins K , Strauch E . Magnetic appendix: an uncommon indication for appendectomy. Cureus. 2022;14:e31096. 10.7759/cureus.31096 36475231 PMC9720090

